# MS analysis of a dilution series of bacteria:phytoplankton to improve detection of low abundance bacterial peptides

**DOI:** 10.1038/s41598-018-27650-4

**Published:** 2018-06-18

**Authors:** Emma Timmins-Schiffman, Molly P. Mikan, Ying Sonia Ting, H. Rodger Harvey, Brook L. Nunn

**Affiliations:** 1 University of Washington, Department of Genome Sciences, Seattle, WA 98195 USA; 2 Old Dominion University, Department of Ocean, Earth, and Atmospheric Sciences, Norfolk, VA 23529 USA; 3 Neon Therapeutics, Boston, MA 02139 USA

## Abstract

Assigning links between microbial activity and biogeochemical cycles in the ocean is a primary objective for ecologists and oceanographers. Bacteria represent a small ecosystem component by mass, but act as the nexus for both nutrient transformation and organic matter recycling. There are limited methods to explore the full suite of active bacterial proteins largely responsible for degradation. Mass spectrometry (MS)-based proteomics now has the potential to document bacterial physiology within these complex systems. Global proteome profiling using MS, known as data dependent acquisition (DDA), is limited by the stochastic nature of ion selection, decreasing the detection of low abundance peptides. The suitability of MS-based proteomics methods in revealing bacterial signatures outnumbered by phytoplankton proteins was explored using a dilution series of pure bacteria (*Ruegeria pomeroyi*) and diatoms (*Thalassiosira pseudonana*). Two common acquisition strategies were utilized: DDA and selected reaction monitoring (SRM). SRM improved detection of bacterial peptides at low bacterial cellular abundance that were undetectable with DDA from a wide range of physiological processes (e.g. amino acid synthesis, lipid metabolism, and transport). We demonstrate the benefits and drawbacks of two different proteomic approaches for investigating species-specific physiological processes across relative abundances of bacteria that vary by orders of magnitude.

## Introduction

Microbes and their physiological processes are the foundation of many ecosystems as they regulate the flow of carbon and nutrients between different reservoirs. Although oceanic primary producers fuel the ecosystem with carbon and nitrogen in the form of dissolved and particulate organic matter (POM), heterotrophic bacteria act as the primary catalysts for remineralizing nutrients back into the ecosystem. This diversity of microbes possesses a bounty of enzymes, allowing them to utilize different energy and carbon sources from a wide range of substrates. To better understand how bacteria function as a collective community, impact their local environments, and control the long-term fate of carbon burial, a variety of ‘omic techniques are now being applied to marine samples (i.e., genomics, transcriptomics, proteomics, metabolomics), providing an unprecedented view of microbial activities across ecosystems^1^. Although there is an expanded body of information on the potential microbes involved in the transformation of organic matter, our current understanding of how *in situ* heterotrophic bacterial communities actively transform, mobilize, and remineralize carbon and other essential elements is only recently being realized.

Metaproteomics is a new approach that has the potential to unravel bacterial-biogeochemical relationships. Due to the tight cellular regulation of protein synthesis and internal degradation, protein abundances reflect the metabolic status and response of a single cell or community of organisms at the time of harvest^2^. When correlated to time specific biogeochemical data, these measurements, set within the context of environmental stimulus, indicate how cells modify their metabolism to acclimate or adapt to the changing chemistry^3^. Although not a direct indication of enzymatic activity, a proteome analysis infers metabolic status and can provide details of protein abundance for processes of interest within an organism or community^4–7^. More direct measurements of protein expression and activity, such as post-translation modifications (PTMs) and enzyme activity, would provide even more accurate interpretations of protein activity and function^8,9^, but methodologies to scale these up to a community level are not developed as they are for full proteome abundance surveys and require significantly more biomass. The detection of protein abundance as a proxy for cellular activity continues to be utilized within the basic science, medical, and environmental fields as it provides an unbiased, rapid survey of the relative presence of thousands of proteins across metabolic pathways.

Historically, discovery-based proteomic techniques have been used to survey the microbial community proteome after fractionation by size^4–7^. The standard MS method for these discovery-style proteomics studies uses data dependent acquisition (DDA; e.g. shotgun or bottom-up proteomics), where peptide ions are selected for fragmentation and tandem mass spectrometry (MS2) based on their initial ion intensity. Resulting from the selection of the 10–20 most intense ions for analysis, this method can fail to detect low abundance proteins^10,11^. Examples across environments demonstrate how detection of relatively low abundance bacterial proteins (due to dynamism of expression level or organism abundance) can be obscured by co-occurring biological material, even though bacteria are present and biologically active^12–15^. Without a method to fully characterize bacterial metabolic processes across oceanic conditions, we cannot fully understand their roles in biogeochemical cycles, which inhibits full and accurate parameterization in predictive models.

A wide range of approaches that facilitate low abundance peptide detection have been investigated, with some techniques applicable to specific sample types (sample preparation^16,17^), while others are more broadly applicable (data acquisition^7,10,18–21^). Here we identify key proteins and peptides of interest from bacteria across a range of abundances with DDA and then use selected reaction monitoring (SRM) to improve the detection range to better include lower abundance peptides. Once peptide targets are identified, SRM assays can then detect and measure a suite of peptides with high sensitivity across a broad dynamic range^22,23^ (e.g., down to attomolar per milligram of total protein). Specifically, this method allows the user to rapidly analyze many bacterial peptides of interest and thus quantify selected microbial metabolic processes^11,24^. This work builds upon DDA characterization of DOM^25–27^ and SRM analyses of a small number of peptides from bacterial cultures and communities^11,24,28–30^ to apply SRM to environments with highly variable abundances of different taxonomic groups.

To investigate detection limits of active microbial processes in eukaryote-dominated systems, a model system comprised of a well-known diatom species, *Thalassiosira pseudonana* (Thaps), and the heterotrophic marine bacterium, *Ruegeria pomeroyi* (Rpom), was used mimic a broad range of bacteria:phytoplankton ratios. A simplified mixture allowed us to demonstrate and define possible variability in bacterial peptide signals among eukaryotic biomass. By tracking this variability, we were able to identify and select bacterial peptides that were below the DDA detection limit through the dilution series and test the ability of SRM to expand the range of detectability in a mixture of proteomes. Because this is a taxonomically complex system, we also emphasize the impact of identical peptide sequences and taxonomically indistinct peptides sequences on the process of SRM assay development.

## Results

### Proteomic differences across the *Ruegeria pomeroyi*: *Thalassiosira pseudonana* gradient

**Figure 1:**
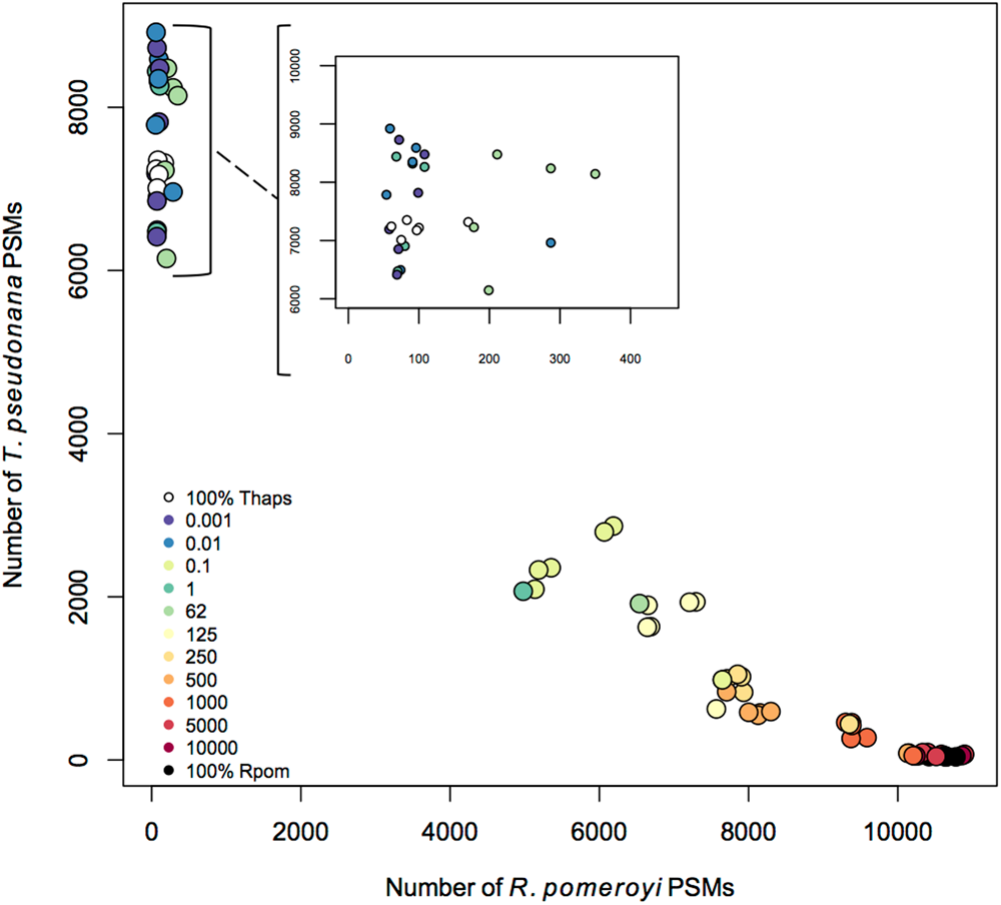
Peptide spectral matches (PSMs) from data dependent acquisition (DDA) of bacterial dilutions in phytoplankton. Points are colored by the cellular ratio of Rpom:1 Thaps cell, indicated in the legends. Number of Thaps peptide spectral matches (PSMs) per injection is plotted against number of Rpom PSMs. The insert shows a detailed view of the upper left-hand corner of the graph.

**Figure 2:**
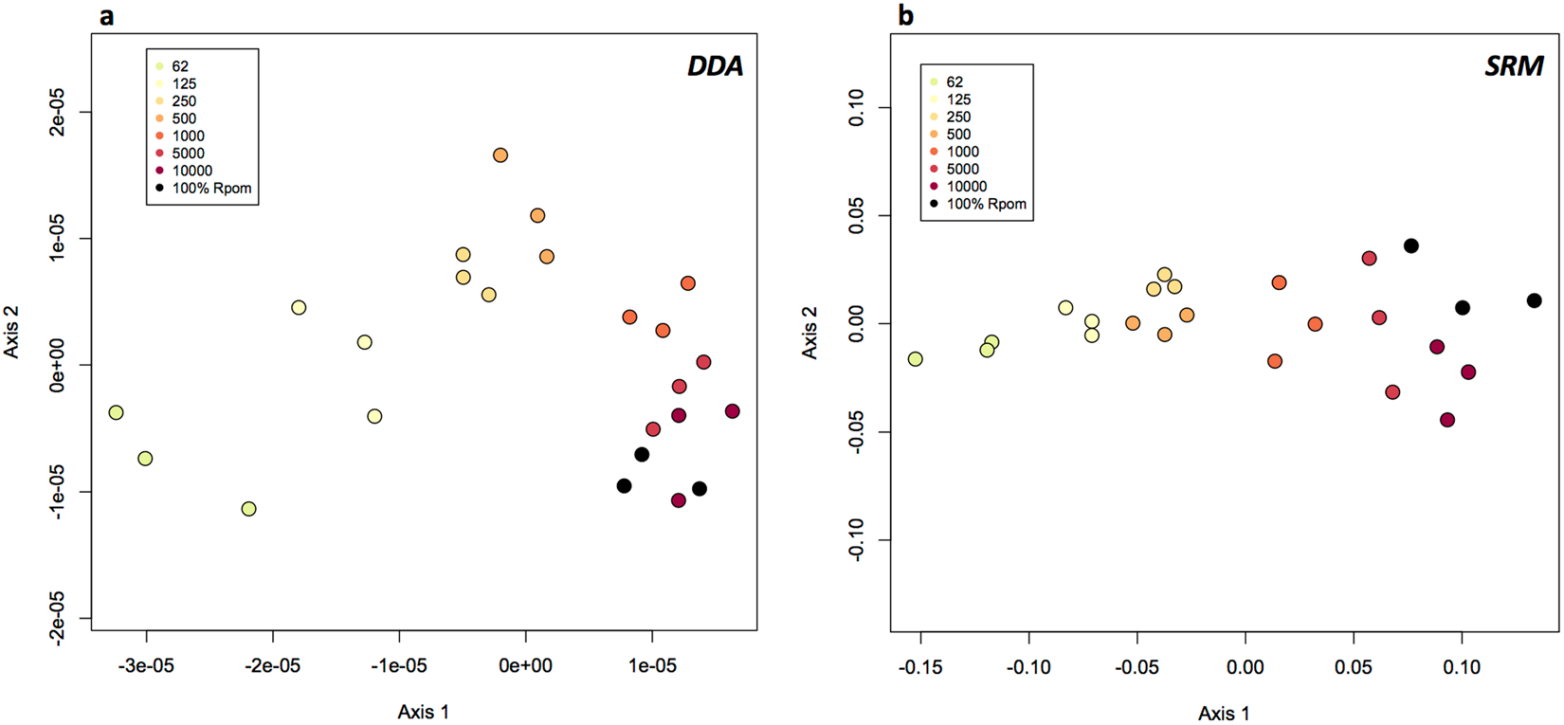
Nonmetric multidimensional scaling plots (NMDS) of proteomic data (normalized spectral abundance factor) from Rpom:Thaps dilutions for DDA (**a**) and SRM (**b**) analyses. Points are colored by cellular ratio Rpom:1 Thaps cell.

Across all bacterial dilutions (*R*. *pomeroyi*: *T*. *pseudonana* 1:1000, 1:100, 1:10, 1:1, 62:1, 125:1, 250:1, 500:1, 1000:1, 5000:1, 10000:1), 3923 proteins were identified using data dependent acquisition (DDA), with 1967 attributed to *Thalassiosira pseudonana* (Thaps) and 1956 attributed to *Ruegeria pomeroyi* (Rpom). The number of Rpom peptide spectral matches (PSMs) increased linearly with the cellular ratio of Rpom cells greater than 62:1 Rpom:Thaps (Fig. 1). Below the 62:1 cellular ratio, most bacterial peptides are undetectable within a DDA experiment. Using non-metric multidimensional scaling (NMDS), there is a suite of 36 Rpom proteins within the DDA data matrix that drives a trend along axis 1, the axis that drives the separation of samples along the cellular dilution gradient (R = 0.8684, p = 0.001; Fig. 2a). These proteins are involved in metal and nucleotide binding. Peptides from these 36 proteins drive this trend in the NMDS because they are under-sampled during MS2 selection, possibly due to low initial relative abundances, HPLC retention time, peptide size, amino acid composition, charge state, and/or hydrophobicity index^31^. Without the collection of a MS2 spectrum, the peptide/protein would remain undetected in a DDA experiment, thus biasing a full proteome analysis and the processes reflected by their expression. This is a recognized limitation in proteomics, however it can be overcome if the researcher begins the experiment with knowledge of what processes, or proteins, they are interested in monitoring.

### Identical peptide sequences in SRM assay development

Peptide sequences can be conserved across taxonomic groups, ranging from species to kingdoms. Typical MS-based bottom-up proteomics (i.e., peptide identification leads to protein inference) followed by database searching identifies an amino acid sequence from an observed tandem mass spectrum. In a taxonomically complex sample, however, a single peptide sequence might correspond to 10 s to 1000 s of species^29^. When searching for the specific signal of a biological or a taxonomic group, it is critical to examine sequence identity in a mixed community sample to eliminate non-discriminatory peptides shared across taxa^29^. The Rpom proteome has 400,708 putative tryptic peptides and the Thaps proteome has 1,887,118 putative tryptic peptides. If all putative peptides were to be detected in an MS experiment, there could be an overlap of 9,328 peptides due to sequence similarity between the two proteomes. However, not all peptides predicted from the genome exist in the digested protein lysates, nor can they all be detected. Across all dilutions 31 peptides with identical sequences were detected in collected tandem mass spectra (Supplementary Table S1). These taxonomically ambiguous peptides are predominantly present in proteins that have basal, highly conserved functions (e.g. ATP synthase, elongation factors, succinyl-CoA ligase).

### Determination of peptide taxonomic specificity

When designing SRM assays for a wider range of environmental contexts, the full taxonomic identity of each peptide must be characterized to determine its taxonomic specificity. Unipept^32^ is a web application that allows users to compare provided lists of peptides to the large non-redundant UniProtKB peptide database to reveal taxonomic specificity based on known peptide sequences. For example, Unipept classifies peptide TVINWAQNAEIFR (from the protein Q5LPJ5, cobalt chelatase) to be identifying of the Rhodobacteraceae family. Most of the Rpom peptides in the SRM assay (n = 28) were found to be specific to the species *Rugeria pomeroyi*, 1 peptide was found across the *Ruegeria* genus, 21 were specific to Rhodobacteraceae (at the family level), 3 are indicative of Alphaproteobacteria (class), 2 were found in all Proteobacteria (phylum), and 6 are found across all Bacteria (kingdom). For the purpose of designing a peptide based SRM assay for metaproteomic samples, a more complete and sample-specific metagenomic-predicted protein database should be investigated for taxonomic specificity since UniPept does not include recent or site-specific protein entries^33^.

### Bacterial biomarker development using targeted proteomics

Targeted proteomics assays were developed from 64 peptides of interest chosen to expand the range of detectability of the bacterial peptides when they were undetectable among the dominating eukaryotic peptides (see Methods section). From the 64 peptides of interest, 305 Rpom peptide transitions (peptide fragments) were detected (Supplementary Table S2). Two hundred seventy-five peptide transitions (90%) were detected across all dilutions in at least one replicate. Additionally, NMDS and ANOSIM revealed a statistically significant difference in peptide biomarker abundance along the bacterial dilution gradient (R = 0.778, p = 0.001; Fig. 2b).

Based on Pearson’s correlation coefficients between SRM peptide transition abundance and bacterial dilution factor, 199 (out of 305 total) transitions had a significant correlation with relative bacterial cell abundance (Pearson’s r ≥ 0.755; Supplementary Fig. S1). These 199 peptide transitions could be detected and used both as biomarkers of bacterial function and relative abundance within a mixed sample in this particular environmental context. The peptide transitions were chosen as representative biomarkers originating from proteins across cellular functions including translation, amino acid and lipid biosynthesis, membrane transport, signaling, vitamin B biosynthesis, and ATP metabolism.

**Figure 3:**
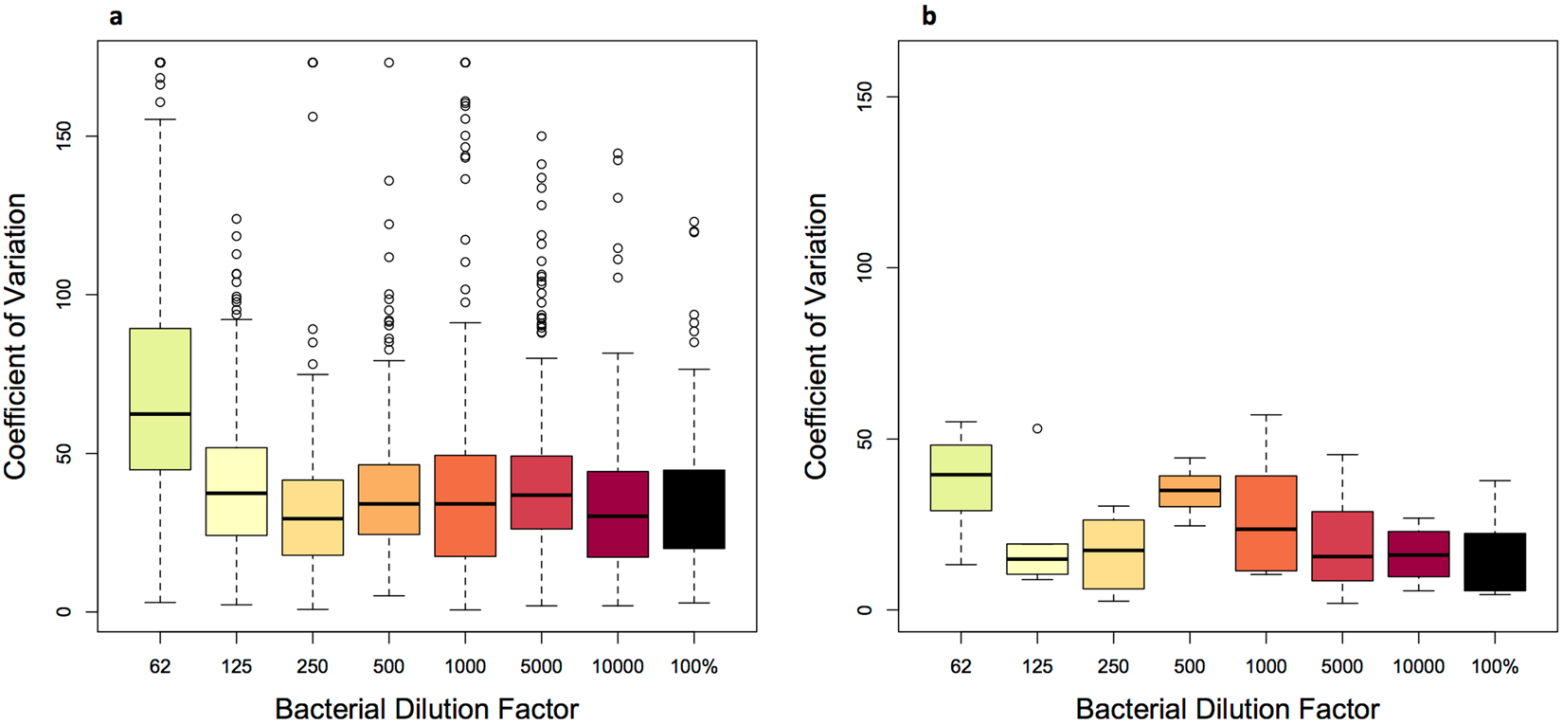
Integrated peak area coefficient of variation values across: (**a**) all peptide transitions (n = 305) for each bacterial dilution and (**b**) 10 transitions with the lowest sum of CV across all samples, representing the most reliable transitions (Supplementary Table S5). These 10 peptide transitions with the lowest CV correspond to 7 peptides from 7 proteins involved in diverse functions (e.g., purine metabolism, fatty acid biosynthesis, transcription). X-axis values correspond to the bacterial dilutions, indicated by cellular ratio Rpom:1 Thaps cell. The boxes represent the upper and lower quartiles of the data distribution; the horizontal black line represents median value; “whiskers” extend to the greatest and least values, excluding outliers; open circles represent outliers (±1.5 times the upper or lower quartiles).

An examination of coefficients of variation (CVs) for the Skyline-derived integrated peak areas for all the transitions finds that peptide and peptide transition selection is critically important and can significantly affect data reliability. A low CV is desirable because it indicates a consistent peptide transition signal, in this case across the integrated peak areas of biological replicates of the same bacterial dilution. In the lowest bacterial abundance sample, CVs were >100% for 19% (n = 55) of the chosen peptide transitions. Samples with higher relative bacterial abundances yielded a trend of improved CV across all peptide transitions considered (Fig. 3). In dilutions of 250:1 (bacteria:diatom) or greater, 1–5.6% of the transitions were reported with high CVs (>100%). Quantification of bacteria-specific peptides was more reliable at greater relative cellular abundances.

## Discussion

The range of bacteria:phytoplankton ratios examined here clearly demonstrate that SRM detects bacterial peptides across a wide range of abundances and physiological processes. We demonstrate how mass spectrometry-based proteomics has the flexibility and selectivity to detect known peptides of individual bacterial species with a lower relative protein abundance living amongst larger volume species with higher relative abundances of proteins (phytoplankton), but this method could also be adapted to other mixed communities. We selected informative biomarker peptides discovered during the DDA analysis to characterize bacterial processes across a wide range of phytoplankton concentrations. These biomarker peptides were then analyzed using SRM, thereby building on existing environmental applications for this pipeline^24,28,29^. In many environments, researchers desire the ability to select and detect specific markers for the presence of an organism within a mixed community. Although SRM is a proven technique in open ocean samples enriched for microbial communities using filtration, environments with low microbial to particle/detrital biomass ratios (e.g., during phytoplankton blooms) offer extra challenges. MS-based methods have the resolution, selectivity, and sensitivity to be an ideal analytical tool for these questions with the added benefit of sequence specificity that can reveal information on biological function and even taxonomy^32^.

The DDA-to-SRM pipeline demonstrated here allows users to first develop testable hypotheses (DDA) on a small number of samples and then rapidly apply them to multiple, complex, mixed community environmental samples. Similar pipelines have been previously applied to explore selected peptides to test hypotheses on specific organism-driven processes and proteins, such as iron preservation^28^, iron limitation^30^, and nutrient stress^24^. The peptides of these targeted proteins were detected at abundances as low as 0.0003–20 fmol/*µ*g protein^28,30^. We expanded upon this foundational work by targeting peptides across a broad range of physiological processes that were technically difficult to detect due to low abundance. These advances show that SRM can be leveraged to interrogate a suite of important physiological processes in species present at low cell counts when peptides specific to the species and process of interest are known. Although experimentally simplified, results demonstrate that SRM can detect peptides in samples with low concentrations of a bacterium that co-occurs with phytoplankton, suggesting that the method could work on unfractionated samples collected during a phytoplankton bloom^34–36^. With this proof of concept, investigators can apply SRM assays to mixed eukaryotic-prokaryotic communities. Assurance of detection and quantification of a signature peptide within a complex sample is increased if the peptide is observed in either DDA or DIA discovery experiments prior to SRM assay development. The workflow can be economized by pooling samples in the DDA or DIA analysis to limit the number of LC-MS/MS analyses and experimentally verifying the presence of high-responding peptides^37^. Although assay development is more streamlined if peptides are first characterized in a preliminary, discovery-based MS step, there will be cases where peptides from a candidate protein of interest are not observed in the discovery phase. These candidate proteins could be selected through genomic predictions, literature discovery, or data mining. Investigators can then utilize one of the many proteotypic calculators that model peptide sequence physiochemical properties to predict high-responding peptide targets for SRM assay development^31^. Additionally, as the available spectral library databases are growing, it is possible to find previous MS experiments that provide charge state, transition lists, and retention times^38,39^. Development of environmental-specific data repositories for this kind of work are currently under construction and will rapidly excel the field^40^. Further work can expand this foundation to more complex, multi-taxa communities.

Taxonomic characterization of peptides in an SRM assay could facilitate our understanding of functional redundancy and specificity across taxonomic groups within a complex bacterial community. We informally demonstrated this potential by analyzing our SRM peptides with Unipept to characterize taxonomic specificity of each peptide. The largest group of peptides are specific to our species of interest, *R*. *pomeroyi*, but if we were to apply our assay to a mixed environmental community we would also glean information from more general taxonomic groups, from the genus to kingdom levels. These results underline the importance of understanding the taxonomic specificity of peptides when selected for an assay of a mixed community, but also the flexibility of this type of analysis. Carefully selected peptides could reveal the extent of functional redundancy and specificity within a natural community, which would significantly augment our understanding of ecosystem function.

In a standard DDA experiment, only the most relatively abundant peptides are selected for full MS2 analysis^41^, however the advantage is that no prior knowledge of protein sequence or detectability by the mass spectrometer are necessary. The detection limitation of DDA is empirically demonstrated here when the peptide signals from bacterial proteins in a mixed prokaryotic-eukaryotic dilution series were reduced when bacterial abundance was below the 62:1 cellular ratio in the DDA dataset (Fig. 1). This ratio (62:1) and below represent some of the typical oceanic concentrations of bacterial cells. Isolation and selectivity of bacteria-specific peptides using SRM significantly improved the detection of targeted bacterial peptides in all experiments from the dilution series. Similarly, targeted analysis detected B12 synthesis peptides that were below the detection of DDA in a Ross Sea bacterial community^11^. A technical limitation of DDA tandem mass spectrometry is the stochastic nature of how ions are selected for tandem mass analysis (i.e., MS2 spectral generation). Because DDA is an analysis that is strictly tethered to the peptide elution profile, co-eluting peptides will interfere with MS2 collection and subsequent identification. As a result, peptides from abundant proteins in the bacteria fraction may decrease in relative intensity as eukaryotic peptides fill the MS1 survey scan. This would lead to fewer, or zero, MS2 collected on those bacterial peptides, ultimately leading to a potential bias in biological data due to protein detectability. In the demonstrated application, DDA or DIA could be applied to a lab culture or sample of a mixed community to detect a broad range of peptides in an unbiased manner to provide guidance in selecting appropriate SRM targets. Subsequently, an SRM peptide assay could be applied across environmental scenarios to capture the dynamism of organism presence and protein expression.

The loss of peptide signal from low abundance proteins in complex mixtures is an analytical challenge that researchers have been trying to resolve for years^16–18,42,43^. Peptides from a candidate protein may be under-sampled in a mass spectrometry experiment as a result of native protein expression, relative abundance of the organism in a mixture, HPLC retention time, or inherent physicochemical properties such as sequence length, amino acid composition, and hydrophobicity. Valuable information regarding bacterial contributions to ecosystem processes across a dynamic range of expression (whether that be the human gut or open ocean) can be lost due to technical limits of detection. Low abundance peptides in DDA experiments can be obscured by peptides from the host of a microbiome^12^ or by the relatively greater abundance of other species within a community^13^. Similarly, in both planktonic and benthic samples of mixed detritus consisting of phytoplankton and bacteria in the Bering Sea, only a few prokaryotic proteins were identified, even though high bacterial counts were observed and bacteria were actively degrading dead phytoplankton^14,15,44^. In these studies, only 2–7 bacteria-specific proteins were detected using DDA, making it difficult to assess bacterial metabolic processes and mechanistic controls, and to quantify the extent of their contribution to overall ecosystem processes. This low detection of bacterial peptides translates to interpretations of bacterial community metabolism based primarily on highly abundant proteins. SRM approaches detect a diverse suite of proteins across abundance ranges that vary by orders of magnitude, including very low intensity peptides such as those that were measured across all samples in this experiment (6 peptides from 2 proteins).

Low quantities of proteins were consistently detected across most dilutions using a targeted approach, however coefficients of variation were high for many of the peptide transitions. As noted by others, this suggests that analytes must be screened for their utility in absolute quantification^45^. It can be difficult to achieve reliable quantification of peptides that occur at relatively low abundance in complex mixtures using MS methods due to co-eluting, interfering peptides or misidentified and misquantified peptide transitions^46^. There is no correlation between high CV and peptide transition retention time, suggesting that retention time does not affect peptide transition stability and that there is little interference from co-eluting peptides in target peptide detection. Additionally, peptide transitions yielding the highest CVs were observed in the sample with the lowest bacterial abundance, suggesting that peptide transition quantification accuracy decreases with the target species relative abundance in a sample. In previous work, CVs for SRM assays on low abundance peptides in single-species protein lysates ranged from 5.4–16.8%^23^. Peptide inter-run variability is important to consider in assay development because a target with high inter-run variability would lead to low confidence peptide quantifications. Decreasing the number of peptides and transitions per MS injection would improve quantifications for low abundance peptides by increasing the dwell time of the triple quadrupole for each transition^22^.

Despite these promising results, SRM is not the panacea to low abundance peptide and protein detection. SRM assays designed for complex environmental communities where species have little available sequence data, can require investment in preliminary detection of peptides using DDA or DIA^47^. Even this preliminary step can be biased, with the several alternative methods now available that allow for the development of SRM assays without the requirement of MS-based peptide detection^48–51^. To detect low abundance peptides using only DDA, sample fractionation should be considered to reduce peptide complexity in MS experiments despite their varied recoveries. Chemical fractionation to remove interfering matrices, depletion of high abundance proteins, multiple filtrations steps, protein size fractionations (including gel separations), and selective antibody removal are all examples that would decrease sample complexity in MS experiments, allowing access to lower abundance proteins for DDA experiments. Additionally, not all techniques are applicable to every system; for example, selective antibody removal^17^ is common in some model systems but would be challenging to apply to complex, uncharacterized systems when interfering, high abundance protein sequences are unknown. Gas phase fractionation within the mass spectrometer with DDA experiments has been shown to dramatically improve the depth of proteome discovery^10,52^. DIA has been reported to improve peptide discovery by 94%^10^, detects many more peptides than DDA, and does not require sample fractionation or enrichment; however, current bioinformatic pipelines for complex communities are less established than for DDA or SRM. DIA does not limit the mass spectrometer to collecting MS2 only on ions with intense precursor signals, as in DDA, and thus dramatically increases the dynamic range of the MS and increases overall proteome coverage by detecting peptides that occur at lower abundances^10,52^. However, due to the multiple injections per sample required to cover the full range of masses, DIA can require significantly more MS time and starting material than SRM, which may not be feasible with environmental samples^37^. Additionally, once an SRM assay is developed, it is imperative to ensure the specificity of the peptide transitions monitored, especially since multiple peptides can co-elute resulting in different fragments within an MS2 selection window. A peptide’s presence in a sample can be verified with total confidence through the inclusion of a synthesized stable isotope labeled peptide of interest. Specific research goals and limitations of experimental design and samples need to be considered when choosing DDA, DIA, or SRM for characterizing a proteome.

In the oceanic ecosystem, many species coexist and compete as they metabolize, degrade and recycle organic material. Standard MS techniques (DDA) can capture proteomic profiles of the most abundant proteins within a system, but since individual microbe protein contributions vary, the desired signal may not be detected. Through applications of SRM to samples containing some realistic cellular ratios of bacteria and phytoplankton, and with peptides previously characterized on a mass spectrometer, we assessed the ability of targeted proteomics to detect selected metabolic processes of an organism present at low cell counts. In DDA mode, the ability of the mass spectrometer to detect bacterial peptides declined with a reduction in relative bacterial abundance; yet targeted SRM analysis reliably detected the metabolic signals of our desired bacterial species of interest across the full dilution series. Although SRM can detect and quantify peptides down to the attomolar level, knowledge of the peptide sequence detectability is required for assay development. This can be obtained with proteotypic peptide calculators that predict detectability based on physio-chemical properties^31,37,53–55^, or experimental determination using DDA or DIA, or the mining of previously published spectral libraries^56^. The reliability of these assays can be determined via a first-round of SRM analysis, with subsequent rounds dedicated to refining the set of peptides included in the assay. These assays could be used to probe microbial metabolic processes across a range of environments to better understand the ecosystem-level transfer of essential nutrients^57^.

## Methods

### Dilution series

The marine diatom *Thalassiosira pseudonana* (Thaps, CCMP1335) was grown in f/2 media^58,59^ with autoclaved and filtered artificial seawater (salinity 30) at ambient room temperature (18–22 °C) under a 13:11 hour light:dark schedule. Diatom growth was monitored by absorbance measurements at 550 nm (Spectronic Educator, Flinn Scientific, Batavia, IL). Cell counts and cellular health were checked throughout the growth cycle with a hemocytometer on an Olympus Optical epifluorescence microscope. The culture was harvested during exponential growth.

The marine heterotrophic bacterium *Ruegeria pomeroyi* (Rpom, NCMA B3) was reconstituted in autoclaved and filtered 0.5 YTSS media^60^ and slowly transitioned into a low carbon (as 0.625 mM glucose) medium over multiple generations. Cultures were grown under axenic conditions at room temperature and bacterial growth tracked by absorbance measurements at 600 nm. The culture was harvested during early stationary phase for experimental mixtures.

To mimic a wide range of oceanic POM samples, a dilution series involving mixtures of Rpom and Thaps was created using different cellular ratios of bacteria:phytoplankton based on previous publications of bacteria and phytoplankton counts in (1) mesocosm experiments^61,62^, (2) before, during, and after phytoplankton blooms^34–36^, and (3) as a function of depth^63^. In nature, these ratios can vary by an order of magnitude^34,35,63,64^ and we extended our dilution ratios to better define the upper and lower limits of mass spectrometry detection within these complex mixtures. Aliquots of cultures at concentrations of 10^8^ cell ml^−1^ (Rpom) and 10^5^ cell ml^−1^ (Thaps) were mixed to yield desired Rpom:Thaps ratios (outlined in Table 1) in triplicate. Samples were subsequently filtered onto 47 mm, 0.2 *µ*m Nucleopore polycarbonate filters (Whatman, Maidstone, UK) to simulate simultaneous *in situ* ocean collections of these mixtures onto a 0.2 *µ*m filter. Once filtered, cells were killed with a 5 ml rinse of cold 10% TCA before the filtered samples were frozen in liquid nitrogen and stored at −80 °C. After protein digestion, additional dilutions were created from these original samples based on calculated cell counts to yield Rpom:Thaps ratios of 1:1000, 1:100, 1:10, and 1:1 for the DDA analysis.

**Table 1:** Cell counts of the bacteria *R*. *pomeroyi* (Rpom) and diatom *T*. *pseudonana* (Thaps) mixtures produced by serial dilution and the calculated ratio of estimated protein contributed from each source (Rpom g protein/Thaps g protein).

#Rpom cells: # Thaps cells	100% Thaps	62	125	250	500	1000	5000	10000	100% Rpom
Rpom cells	0	2.08 × 10^9^	2.08 × 10^9^	2.08 × 10^9^	2.08 × 10^9^	2.08 × 10^9^	2.08 × 10^9^	2.08 × 10^9^	2.08 × 10^9^
Thaps cells	8.33 × 10^6^	3.33 × 10^7^	1.67 × 10^7^	8.33 × 10^6^	4.17 × 10^6^	2.08 × 10^6^	4.17 × 10^5^	2.08 × 10^5^	0
Protein ratio		4.9	9.8	19.7	39.3	78.8	393.0	788.0	

The Rpom cellular protein content is estimated from^46^ and Thaps cellular protein content is estimated from^84^.

### Protein extraction

Proteins were extracted from filters by shaking the filters suspended in 500 *µ*l of 6 M urea in a bead beater with no beads (repeat 3 times:1 min shaking; ice 5 minutes). After removing the filters from the liquid, cells were lysed using a sonicating probe^3^. This method removed an average of 42% (range: 9–94%) of total proteins from the filter, determined by amino acid quantification (Supplementary Methods and Supplementary Table S3).

Protein concentrations were measured using the BCA assay (Pierce, Thermo Fisher Scientific), following the manufacturer’s protocol. All samples were analyzed in triplicate and concentrations were averaged for a final protein concentration. Digestions of 100 *µ*g of protein were completed following^3^.

### LC-MS/MS and protein inference: DDA

**Figure 4:**
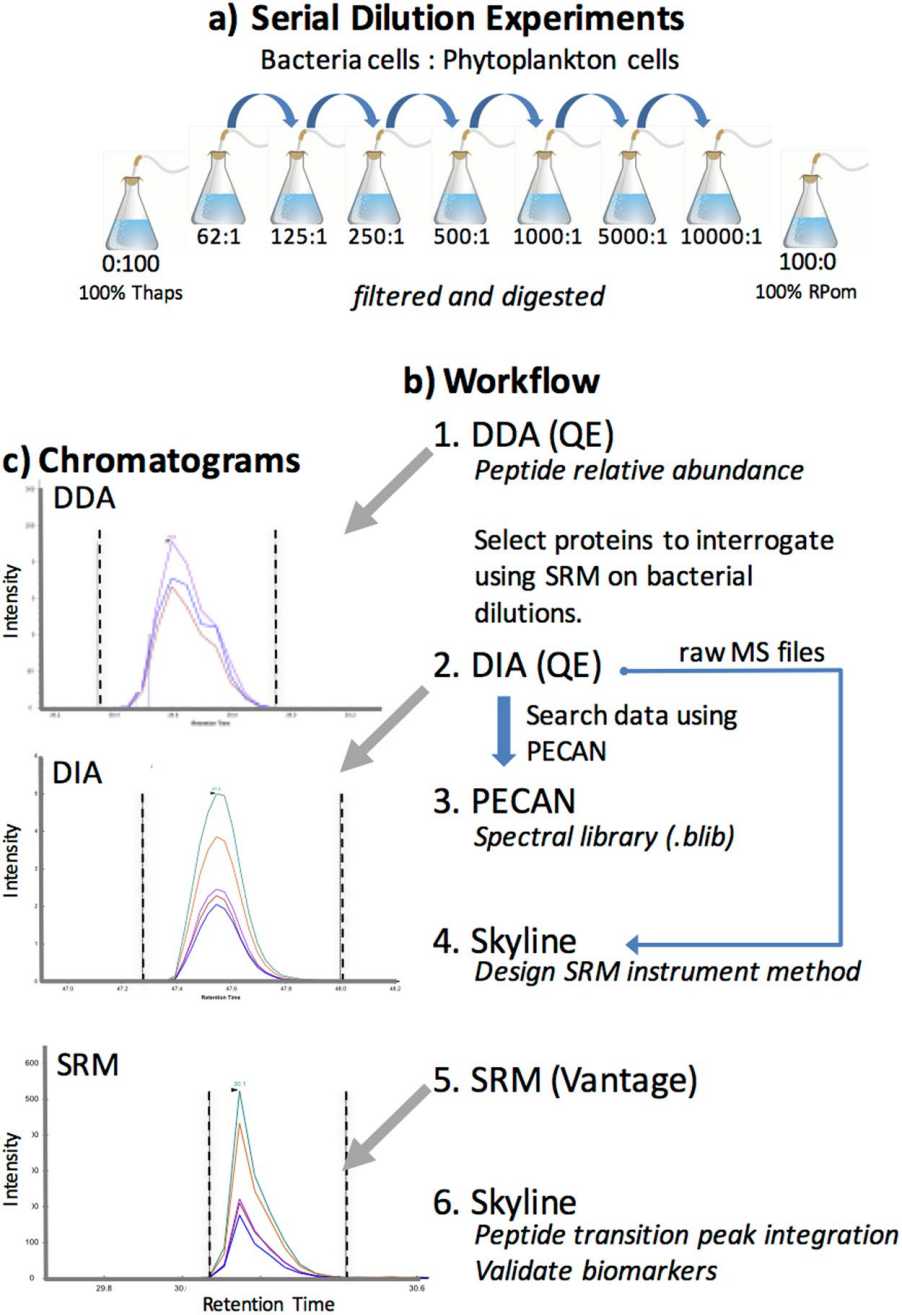
Illustration of experimental setup and workflow for mass spectrometry data acquisition and analysis. (**a**) Serial dilutions were completed using bacterial cells (RPom) as the diluent (see text). Dilution was based on cell counts to achieve cellular rations of Rpom (*R*. *pomeroyi*) to Thaps (*T*. *pseudonana*). Each serial dilution was then lysed and proteins were digested prior to MS experiments. (**b**) MS experimental workflow: 1. Data dependent acquisition (DDA) was performed on the Q-Exactive-HF (QE) to assess the limit of detection for a standard, discovery-driven proteomics experiment. 2. Data independent acquisition (DIA) was also completed on the QE to create spectral libraries for selected reaction monitoring (SRM) method development. 3 & 4. These spectral libraries were analyzed with PECAN and Skyline was used to select optimal transitions and to design an instrument method for SRM analyses. 5. SRM was completed on the TSQ Vantage for 309 bacterial peptide transitions. 6. Peptide transition detection and quantification was performed in Skyline. (**c**) The chromatograms of peptide IPSAVGYQPTLATDMGAMQER (from protein Q5LNP1) were collected using the 3 different MS approaches (DDA, DIA, and SRM) on bacterial dilution 5000:1. Black vertical lines indicate peak integration boundaries, and colored peaks represent the different transitions (i.e. peptide fragments) collected.

Liquid chromatography coupled with tandem mass spectrometry (LC-MS/MS) was completed on a Q-Exactive-HF (QE: Thermo Fisher Scientific) in technical duplicate analyses for each sample using data dependent acquisition (DDA) on the top 20 precursor ions (Fig. 4). The analytical column was 20 cm long and packed in house (3 *µ*m C18; Dr. Maisch) with a 3 cm long trap (3 *µ*m C12; Dr. Maisch). Peptides were eluted using a 5–35% ACN gradient over 60 minute at 300 nl/min flow rate. MS1 ions were collected in the scan range of 400–1400 *m/z*. Automatic gain control threshold was set at 1 × 10^6^ for MS1 and 5 × 10^4^ for MS2 and dynamic exclusion of 30 s was used for MS2. The mass spectrometry proteomics data have been deposited to the ProteomeXchange Consortium via the PRIDE partner repository^39^ with the dataset identifier PXD004799 (http://www.proteomexchange.org/).

### Database search parameters

Completed proteomes for *Thalassiosira pseudonana* and *Ruegeria pomeroyi* were downloaded from Uniprot (7/2013; www.uniprot.org). These databases were concatenated with 50 common contaminants, yielding a protein database of 17,395 proteins. To assign spectra to peptide sequences, correlative database searches were completed using Comet v. 2015.01 rev. 2^65,66^. Comet parameters included: Trypsin enzyme specificity, semi-digested, allowed 1 missed cleavage, 50 ppm mass tolerance, cysteine modification of 57 Da (resulting from the iodoacetamide) and modifications on methionine of 15.999 Da (oxidation). Minimum protein and peptide thresholds were set at *P* > 0.9 on Protein and Peptide Prophet^67^. Protein inferences from the whole-cell lysates were accepted by ProteinProphet if the thresholds noted above were passed, two or more peptides were identified, and at least one terminus was tryptic^68–70^. Normalized spectral abundance factor (NSAF) was calculated^71^ for all inferred proteins^72^.

### Proteomic differences across Rpom:Thaps gradient

Non-metric multidimensional scaling (NMDS) in the vegan package^73^ in R v. 3.2.3^74^ was applied to assess tightness of technical replicates (Supplementary Fig. S3), excluding the 100% Thaps sample. Technical replicate analyses of individual samples showed consistent proteome characterization so spectral counts were averaged across technical replicates to calculate final NSAF and for NMDS and ANOSIM analyses in the vegan package in R.

The Rpom proteins that had significant loadings (p = 0.00099 and loading >0.99) along the NMDS axis that differentiates the samples based on ratio of Rpom:Thaps (Supplementary Table S4) were analyzed for enrichment of specific biological processes in DAVID v. 6.7^75,76^ using the Rpom proteome as the background protein list. These are proteins that are increasingly difficult to detect with DDA methods when bacteria are at relatively low abundance.

### *In silico* analysis of peptide sequence identity and taxonomic specificity

The Thaps and Rpom complete proteomes were digested *in silico* using the Protein Digestion Simulator v. 2.2.5350.26597 from PNNL (omics.pnl.gov) to determine if there would be peptide sequence homology between organisms. The following settings were used: Minimum fragment mass = 400, maximum fragment mass = 6000, minimum residue count = 5, max missed cleavages = 3, hydrophobicity mode = Hopp and Woods. This created two files, one containing putative Thaps tryptic peptides and the other containing putative Rpom tryptic peptides.

Rpom peptides selected for SRM analysis (see below) were compared to all known bacterial peptide sequences in Unipept^32,77^, which searches peptide sequences against the entire UniProt database, on February 13, 2017. This analysis gives the taxonomic specificity of each peptide, i.e. if a peptide is species-specific or found across bacterial taxa at a higher taxonomic level.

### LC-MS/MS: DIA, targeted proteomic assay development, and SRM

Targeted proteomics assays were developed and tested to determine at what point in the dilution steps the bacterial peptides were undetectable among the dominating eukaryotic peptides. Based on the DDA analysis of the bacterial dilution series, peptides in the following categories were selected for targeted assays: (1) peptides present across biological replicates and dilutions, (2) unique peptides identified only in low Rpom:Thaps dilution (i.e., phosphate-specific transport system (Q5LS18) and ABC transporter, ATP-binding (Q5LLS4)), (3) peptides that drive the differences observed in the NMDS (see Methods, Proteomic differences across Rpom:Thaps gradient). These categories yielded an assay of 64 peptides derived from 24 proteins (Supplementary Table S2). Predetermined peptides with identical sequences between the Rpom and Thaps proteomes were not present in the list of peptides of interest for targeted proteomics.

The three technical replicates from the DDA experiment for the dilution of 5000 Rpom: 1 Thaps cell were pooled in equal quantities to create two new technical replicates for data independent acquisition (DIA) on the QE (Thermo). Each sample included a spiked-in internal quality control peptide standard (375 fmol Peptide Retention Calibration Mix; Pierce, hereafter referred to as “QC”). Sample injections for all DIA experiments included 1 *µ*g protein plus the internal standard in a 2 *µ*l injection. DIA experiments were completed using a 27 cm analytical column with a 3 cm pre-column (3 *µ*m C18; Dr. Maisch). Technical replicates were collected in 4 *m/z* isolation width windows in 125 *m/z* ranges (400–525, 525–650, 650–775, 775–900)^10^. For each method, a gradient of 5–80% ACN over 90 minutes was applied for peptide spectra acquisition. Raw data can be accessed via ProteomeXchange (http://www.proteomexchange.org/) under identifier PXD004758.

To generate spectral libraries for targeted method development, Peptide Centric Analysis was completed with the software program PECAN^78^. Input files included the list of peptides generated for targeted proteomics, as described above, and the mzML files generated from the raw DIA files using MSConvert^79^. PECAN correlates a list of peptide sequences of interest with the acquired DIA spectra to locate the peptide-specific spectra within the acquired DIA dataset.

The PECAN.blib output file was then imported into Skyline daily v. 3.5.1.9706^80^ for targeted method development. The targeted method development workflow, including screenshots, can be found in Supplementary Methods. Peptide transitions are defined as the reproducible fragments of peptides that are produced during the MS2 scan in a mass spectrometer^81^. Peptide transitions were selected if peak morphology was uniform and consistent across the MS2 scans for both technical replicates. Peptides were selected for targeted analysis if they had >3 high quality transitions and >3 peptides per protein. Only 4 transitions per peptide were selected for targeted analysis and no more than 3 peptides per protein were selected. The final list consisted of 334 transitions (based on manual protein selection) and this transition list was divided among two method files for the final SRM analyses (Supplementary Table S2). The Skyline document used to make the SRM assay is freely available at Panorama: https://panoramaweb.org/labkey/oceanbact.url.

Selected reaction monitoring (SRM), was completed on a Thermo Vantage for all bacterial dilution samples in Table 1. Samples were prepared as described above for DIA (1 *µ*g of protein per 3 *µ*l injection), and each sample was analyzed individually on the Thermo Vantage. New C18 trap (2 cm) and C18 analytical columns (27.5 cm) were used and each sample was analyzed in two MS experiments to cover the entire peptide transition list (n = 334). Raw data can be accessed in the PeptideAtlas (http://www.peptideatlas.org/PASS/PASS00917) under accession PASS00917.

Acquired SRM data were analyzed in Skyline (https://panoramaweb.org/labkey/oceanbact.url). Peptide transition MS2 peaks were quantified using peak area integration across all samples. Peak presence was determined based on consistency of retention time (verified by spiked in QC peptides) and peak morphology. Relative retention times for QC and bacterial peptides were correlated between DIA and SRM experiments with an R^2^ > 0.99 (Supplementary Fig. S2).

All peptide transition peak intensities were exported from Skyline for analysis. QC transitions were assessed for consistency across runs by calculating the coefficients of variation (CVs) of transition peak area across injections in the raster package^82^ in R v. 3.2.3^74^. The eight QC transitions with the lowest CV (<40) were used for inter-run normalization. Peak intensities for 305 bacterial transitions were normalized by dividing by the averaged intensities for 8 QC transitions within a given run. Normalized peak intensities were analyzed using NMDS and ANOSIM, as described above for DDA. Pearson’s r and the critical r value were calculated in R v. 3.2.3^74^ for the correlation between peptide transition peak intensity and bacterial dilution factor. A heatmap of average peptide transition peak intensities for each dilution above the cut-off of Pearson’s critical r was constructed in pheatmap^83^ in R, with rows (transitions) and columns (dilutions) clustered using Euclidean distance and the average clustering method. Proteins were annotated with Gene Ontology terms using the UniProt Retrieve/ID mapping tool (uniprot.org).

### Data availability

The datasets generated during and/or analyzed during the current study are available in the repositories ProteomeXchange Consortium via the PRIDE partner repository with the dataset identifier PXD004799 for DDA data and PXD004758 for DIA data; Peptide Atlas for SRM data (http://www.peptideatlas.org/PASS/PASS00917) under accession PASS00917; and Panorama for Skyline documents (https://panoramaweb.org/labkey/oceanbact.url). Other data generated or analysed during this study are including in this published article (and its Supplementary Information files).

## Electronic supplementary material

Supplementary Methods

Supplementary Figures S1-S3

Supplementary Tables S1-S5
