# Supplementary material for: MS analysis of a dilution series of bacteria:phytoplankton to improve detection of low abundance bacterial peptides
Source: Sci Rep. 2018 Jun 18;8:9276. doi: 10.1038/s41598-018-27650-4 (PMC6006377; doi:10.1038/s41598-018-27650-4)

Supplementary Methods for

MS Analysis of a Dilution Series of Bacteria:Phytoplankton to Improve Detection of Low Abundance Bacterial Peptides

Emma Timmins-Schiffman

Molly P. Mikan

Ying Sonia Ting

H. Rodger Harvey

Brook L. Nunn

The first page after the title page contains the experimental procedure for quantification of amino acids extracted from PC filters (for proteomics) and of the amino acid fraction remaining on the filter. The next pages contain a tutorial for creating SRM assays in Skyline using DIA data analyzed in PECAN. Permission to use the Skyline logo has been granted by the copyright holder, Brendan MacLean (University of Washington).

***Methods for quantification of amino acids in cells collected on polycarbonate filters***

After urea extraction, sonication, and rinsing with NH_4_HCO_3_ (see Methods), the extracted solution was split to perform amino acid analysis (1/4 of the volume) and proteomics analysis (3/4 of the volume). For amino acids, samples were defrosted and brought to dryness in a speedvac. Individual amino acids were identified and quantified by gas chromatography mass spectrometry (GC-MS) using the EZ:faast method (Phenomenex). The EZFaast method was used to quantify the sum of amino acids that could be extracted from filters with urea as well as the amount remaining on the polycarbonate filter after removal of biological material for proteomics with subsequent hydrolysis. Amino acids in both the extract and remaining on the filter were subject to acids hydrolysis for 20 hours at 110°C with 6M HCl amended with ascorbic acid as an antioxidant. Norvaline (10-300 µl) was added to each sample as an internal standard.

Following derivatization by EZFaast reagents, samples were analyzed using GC/MS as described in Moore et al. (2012). Briefly, amino acids quantified using an Agilent 6890 GC with samples injected at 250^o^C and separated using a DB-5MS (0.25 mm ID, 30 m) GC column with hydrogen as the carrier gas. For amino acid identification the GC was coupled to an Agilent 5973 mass spectrometer run under the same conditions. Bovine serum albumin (BSA) was analyzed in parallel to correct for responses among individual amino acids and calculation of molar ratios. Amino acid concentrations were summed across individual amino acids and corrected for the split in volumes used.

***Selected Reaction Monitoring Workflow (8 steps)***

Additional Tutorials:

Skyline targeted method development - <https://skyline.gs.washington.edu/labkey/wiki/home/software/Skyline/page.view?name=tutorial_method_edit>

Pecan - <https://www.evernote.com/shard/s347/sh/edcb06ab-d008-418f-b28f-52f6614f1c39/2984ab55f427fcfe>

1. Use Pecan to create a library for targeted method development in Skyline.

Input files needed for Pecan

- Raw data files converted to mzML files and a file with a list of paths to these files
- Bacground proteome, if not already included in Pecan
- List of peptides of interest and a path to this list
- List of isolation windows

Run pecanpie to prepare files for Pecan

- Pecanpie –o [name of directory to be created] –b [name of background proteome] –n [name of blib file to be created] --isolationSchemeType BOARDER --pecanMemRequest 10 [list of mzML paths] [list of peptide list paths] [isolation scheme list]

Run Pecan

- Navigate to directory created by pecanpie
- Submit Pecan jobs: ./run_search.sh

Output from Pecan is used directly in Skyline.

1. Import spectral library in to Skyline.

Settings > Peptide Settings > Library

- Click “Edit List”, then “Add” if your library is not already in the list


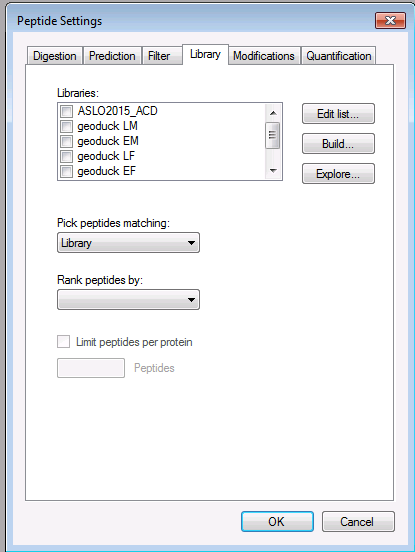


Name your library and select .blib file (generated in Pecan).

After clicking “OK” select correct library from list.

Select “Library” under “Pick peptides matching”


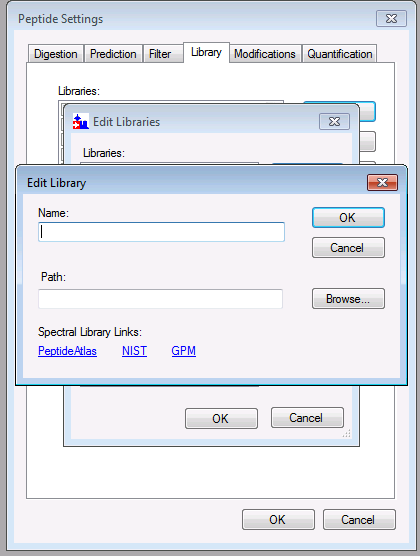


Name of library

Select .blib file

1. Add background proteome to Skyline.

The background is the fasta version of the background proteome from Pecan (since we analyzed a species not included in Pecan’s known list).


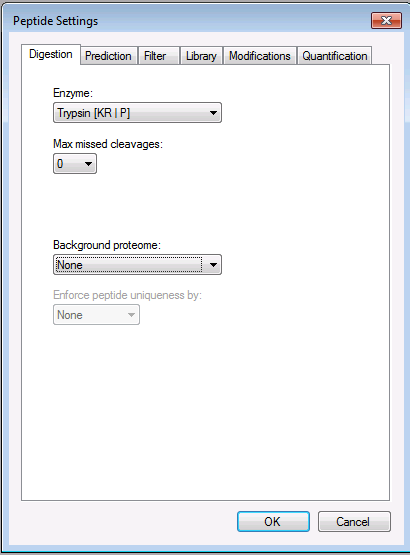


Settings > Peptide settings > Digestion

- - Select “Add” under “Background proteome”

Name background, click “Create” under “Proteome file” to choose where you will save the background.

Click “Add file” under “FASTA files” and select your background proteome fasta file.


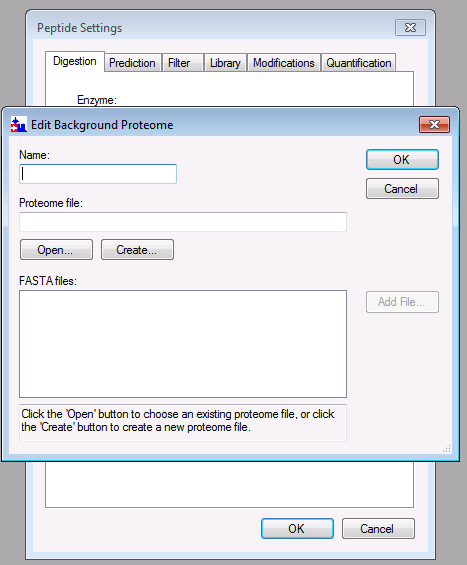


Under Prediction tab, make sure “none” is selected for retention time predictor.

Background name

Background fasta

1. Populate the target analyte tree.


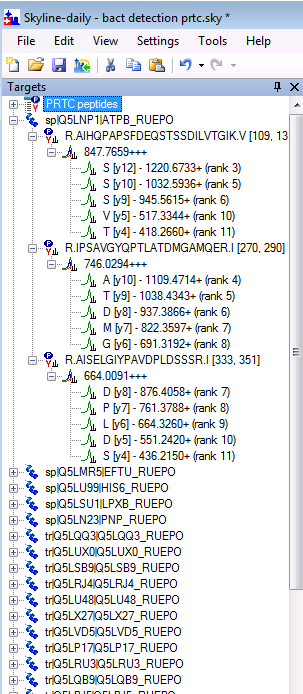


Open the background proteome fasta file and copy all protein sequences.

Paste the sequences into the lefthand, long window in the main Skyline view.

Skyline will keep the proteins, peptides, and transitions that match what it finds in the library you gave it.

1. Adjust transition settings in Skyline.

Settings > transition settings > Filter

- - Precursor charges: 1,2,3,4,5
  - Ion charges: 1 (this is the prevalent fragment, additional charges will increase interference)
  - Ion types: y,p (including b ions for Q-Exactive data will increase noise)
  - Product ions: from ion 3 to last ion -2


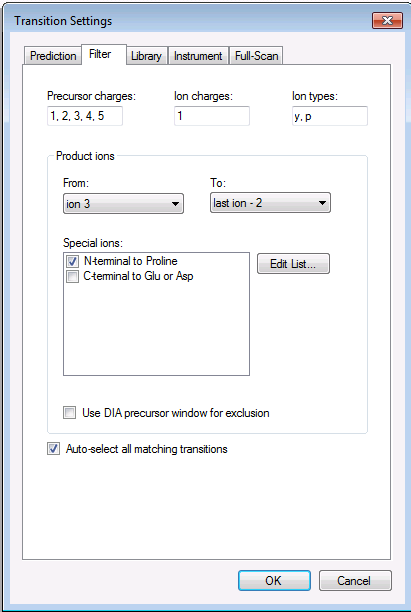


Settings > transition settings > Library

- - Ion match tolerance: 0.5 m/z
  - Unselect “if a library spectrum is available, pick its most intense ion”


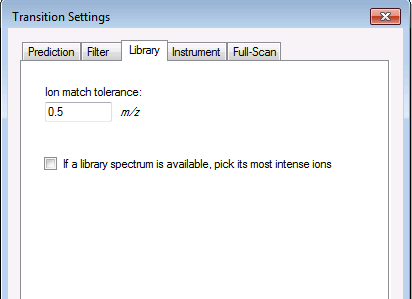


Settings > transition settings > Full Scan

- - MS1 filtering
    - Isotope peaks included: count
    - Precursor mass analyzer: orbitrap (instrument specific)
    - Resolving power: 60,000 at 200 m/z (experiment specific)
  - MS/MS filtering
    - Acquisition method: DIA (experiment specific)
    - Product mass analyzer: centroided (use with automated peak detection)
    - Mass accuracy: 15 ppm (experiment specific)
    - Isolation scheme: copy and paste list of isolation targets (experiment specific)
  - Retention time filtering
    - Use scans within 1 minute of MS/MS IDs


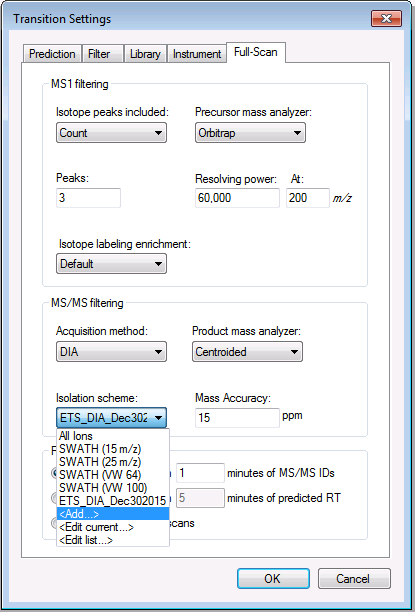


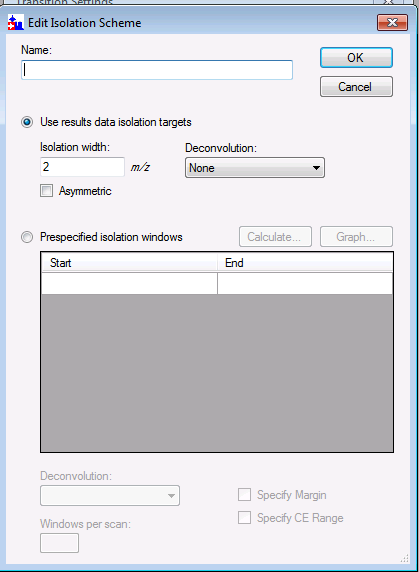


Paste isolation windows

Isolation scheme name

1. Import raw DIA data into Skyline.

Organize your raw files in a directory under a main directory called “raw” with sub-directories for each sample/organism that contains all files for that sample.

In Skyline, File > Import > Results. Select “Add multi-injection replicates in directories”


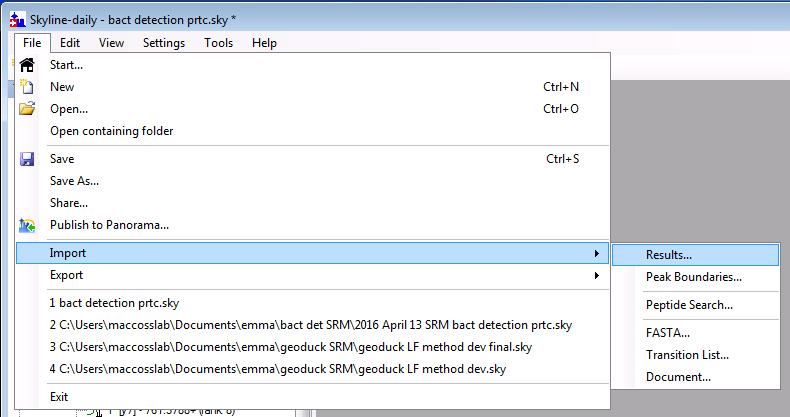


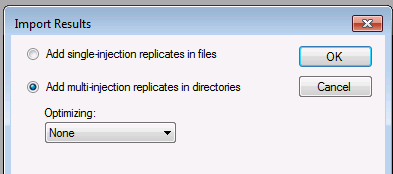


Select the directory of raw files and Skyline will recognize subfolders.

1. Refine selection of transitions for targeted method.

This will depend on the goal for your project. Here are the parameters that I used, with a goal of creating a list of <400 transitions.

Remove all precursor ions.

Remove a protein if:

- It has <3 peptides

Remove a peptide if:

- - It has <4 transitions
  - There are >3 peptides per protein and it is one of the lower quality peptides

Remove a transition if:

- - There is not a clear, single peak in both technical replicates collected in DIA
  - There are >5 transitions per peptide and it is one of the lower quality transitions

8. Export transition list from Skyline to paste into an instrument method.


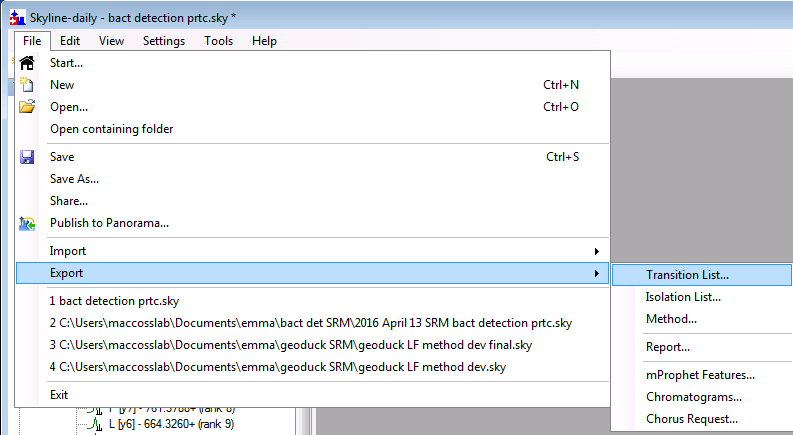


File > Export > Transition List

- Thermo instrument type
- Single method
- Paste transition list into triple quadrupole method


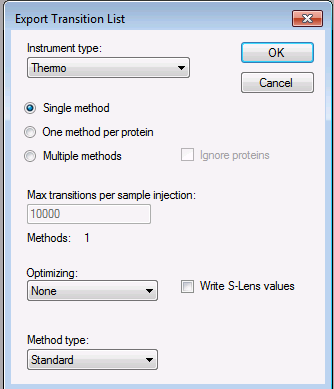

Supplement: Supplementary file 1 — Supplementary Methods [file 41598_2018_27650_MOESM1_ESM.docx]
