# Supplementary material for: MS analysis of a dilution series of bacteria:phytoplankton to improve detection of low abundance bacterial peptides
Source: Sci Rep. 2018 Jun 18;8:9276. doi: 10.1038/s41598-018-27650-4 (PMC6006377; doi:10.1038/s41598-018-27650-4)

MS Analysis of a Dilution Series of Bacteria:Phytoplankton to Improve Detection of Low Abundance Bacterial Peptides

Emma Timmins-Schiffman

Molly P. Mikan

Ying Sonia Ting

H. Rodger Harvey

Brook L. Nunn

Figure S1. Heat map showing the log(x+1)-transformed, QC intensity-normalized MS2 peak intensities for the 199 peptide transitions that were significantly correlated with bacterial cell relative abundance. Rows (transitions) and columns (bacterial dilutions, indicated by cellular ratio Rpom:1 Thaps cell) are clustered based on Euclidean distance, using the average clustering method.

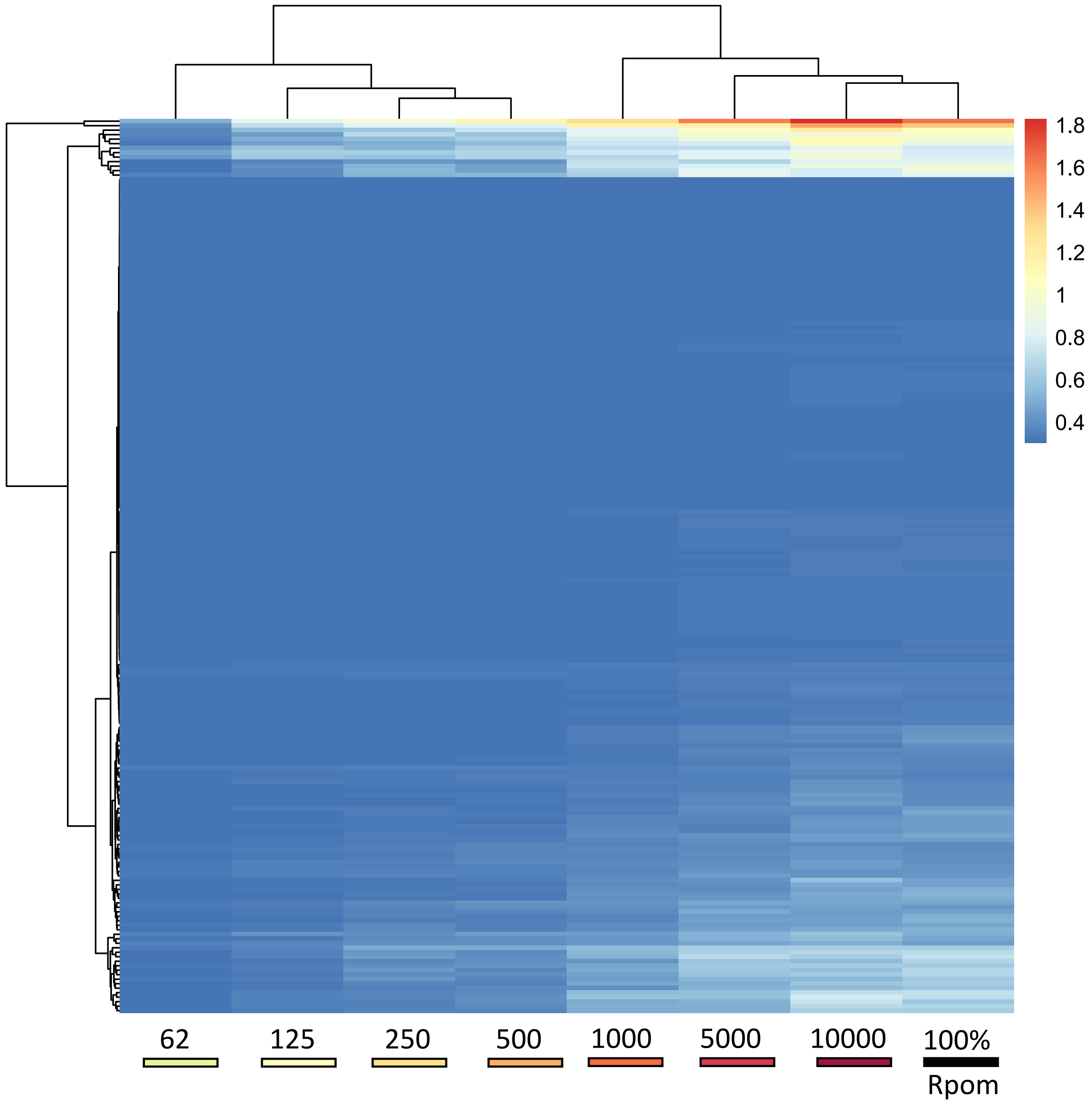

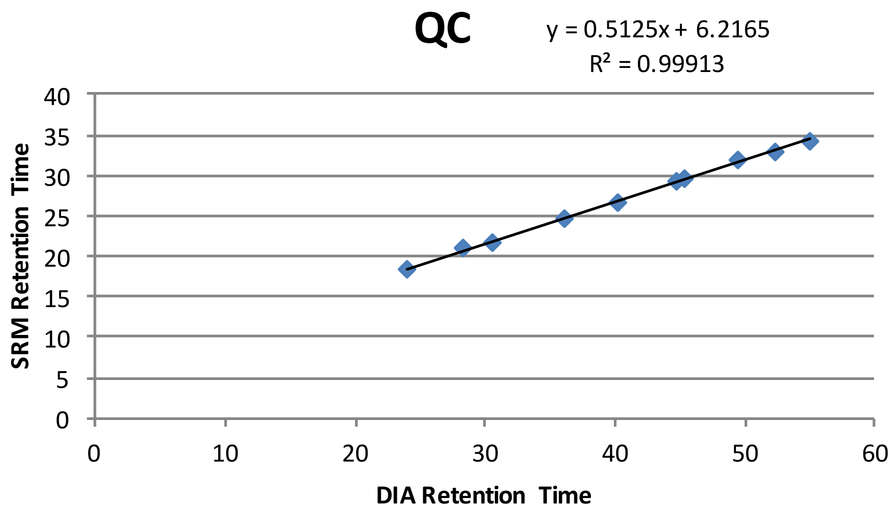

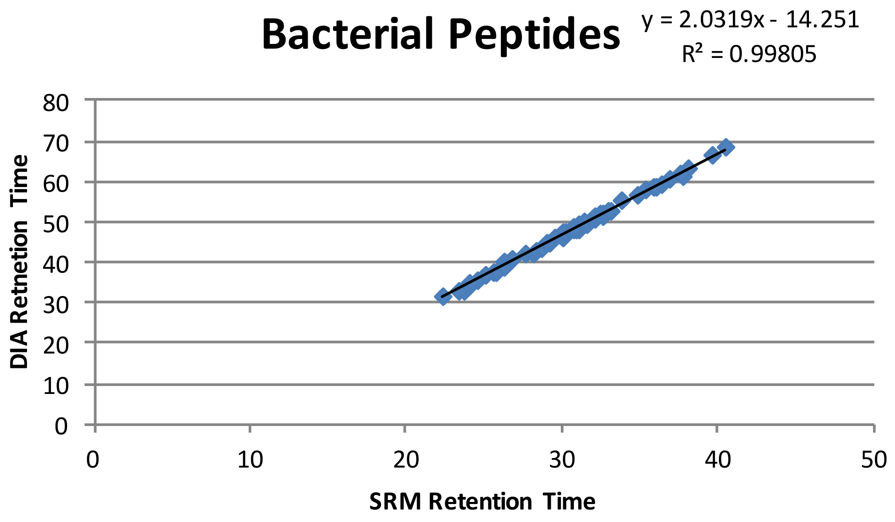

Figure S2. Correlations of retention times between the same peptides identified in Skyline for the DIA and SRM datasets. Plots are presented for the PRTC QC peptides separate from the bacterial assay peptides.

Figure S3. NMDS of non-combined technical replicates for DDA analysis. Technical replicates showed similar proteomic profiles and so were combined for further analysis.

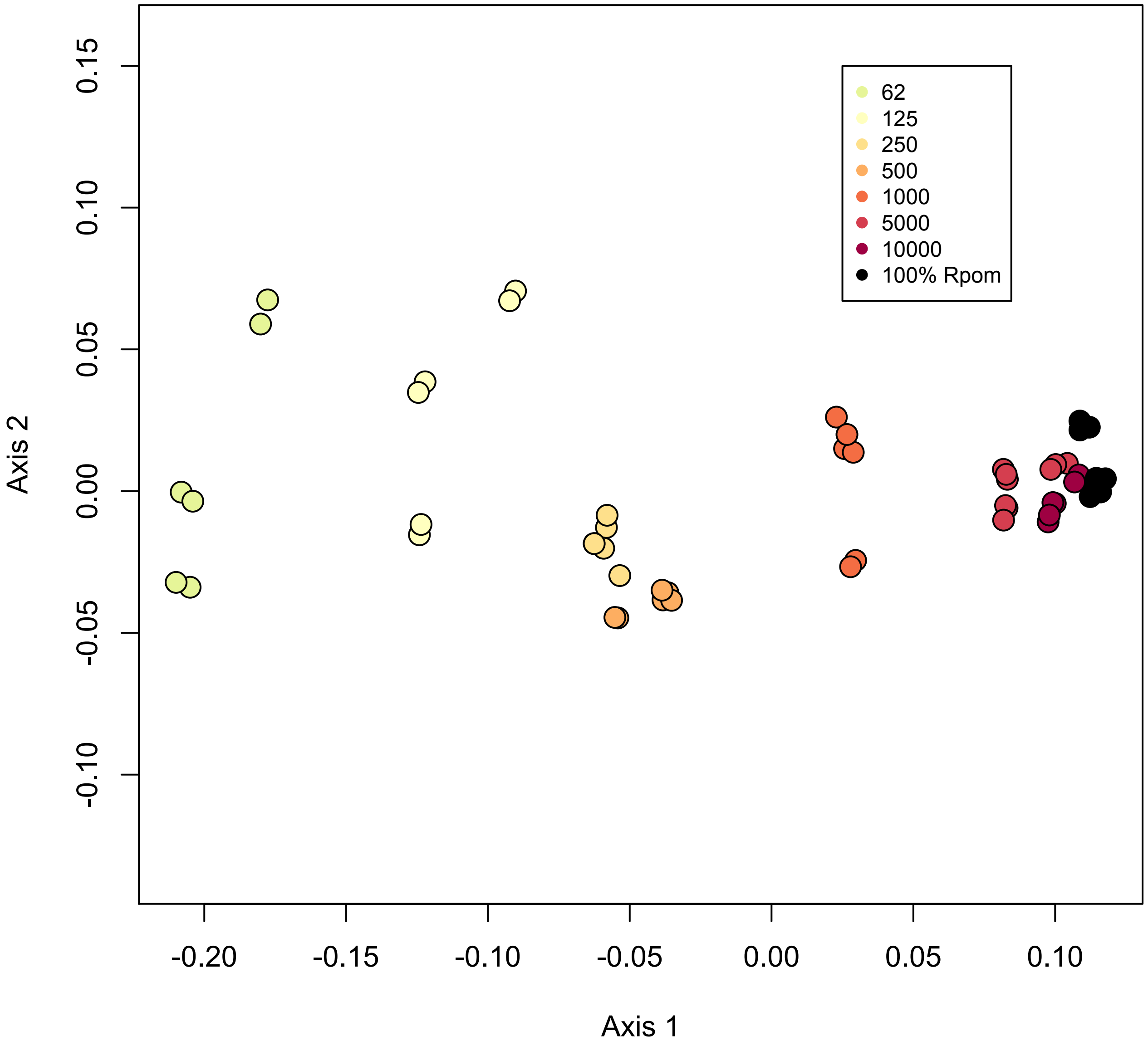

Supplement: Supplementary file 2 — Supplementary Figures S1-S3 [file 41598_2018_27650_MOESM2_ESM.pdf]
